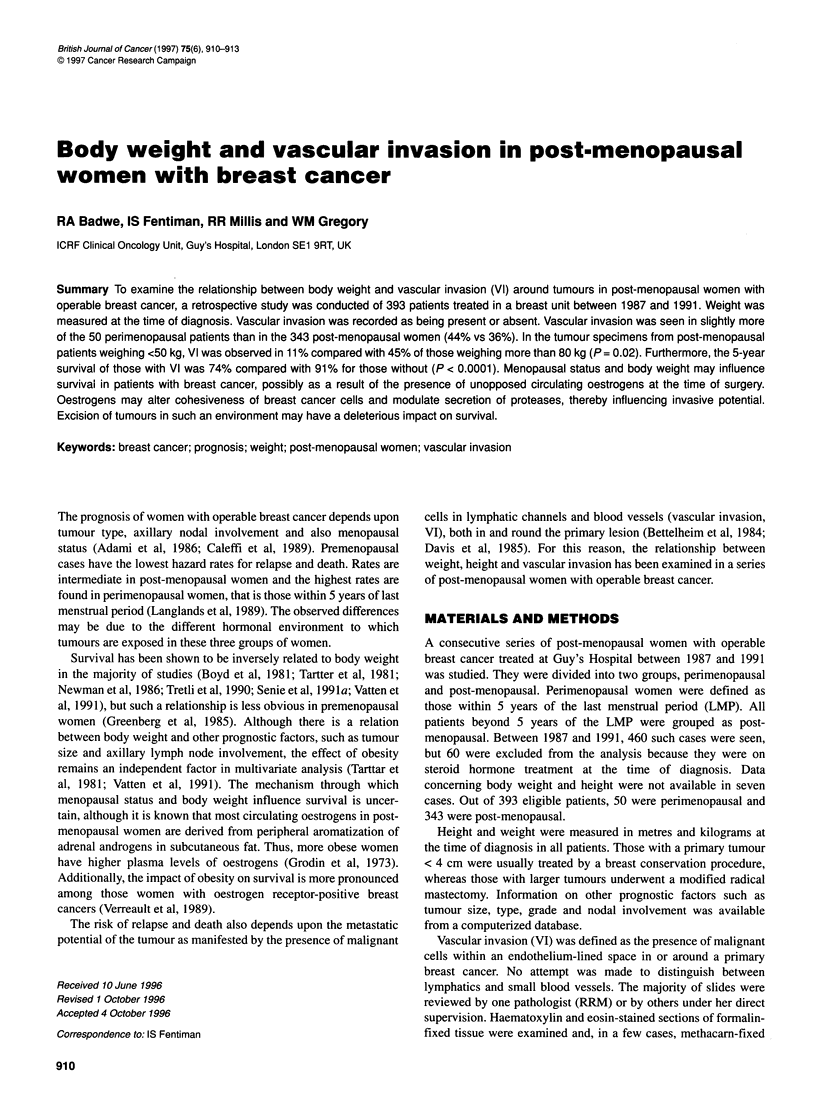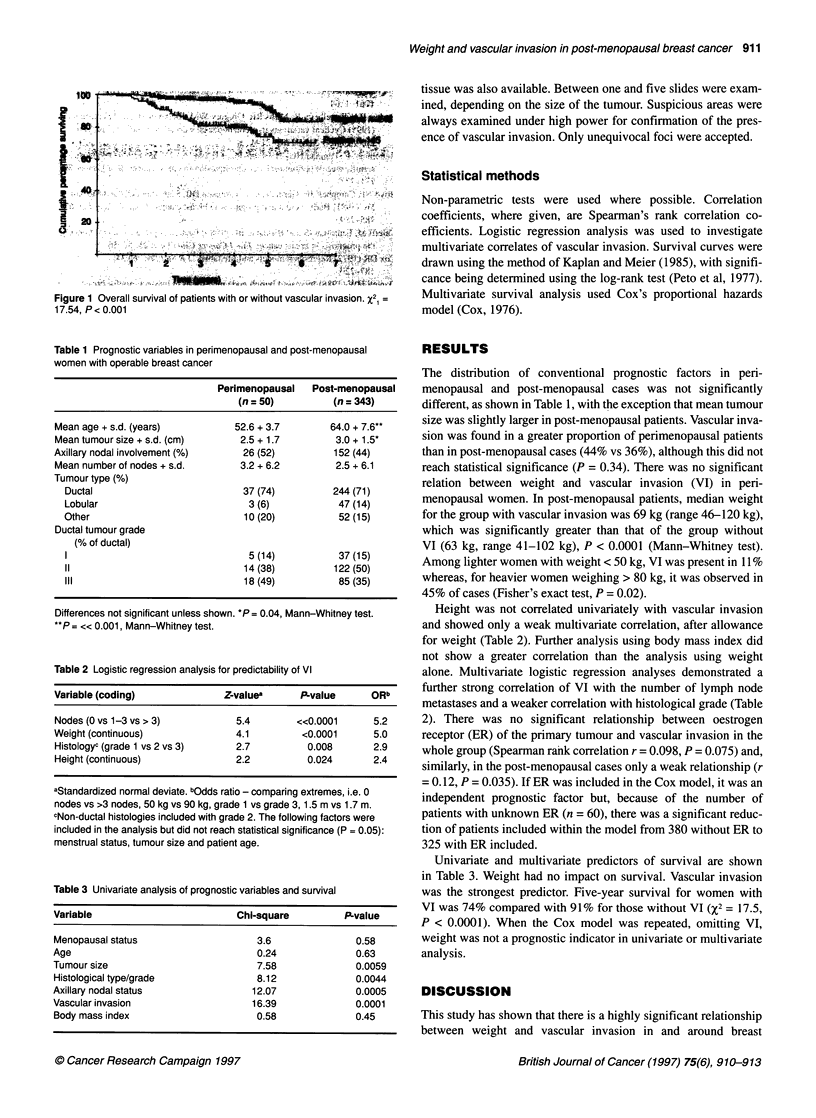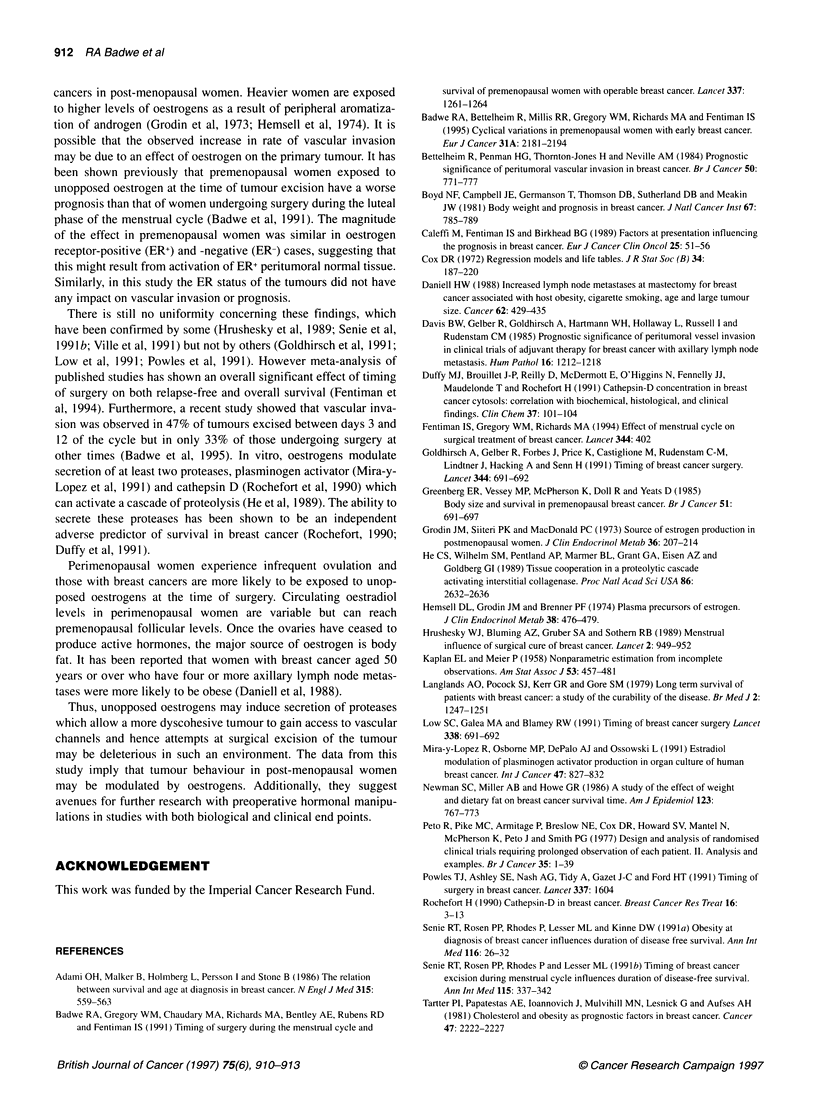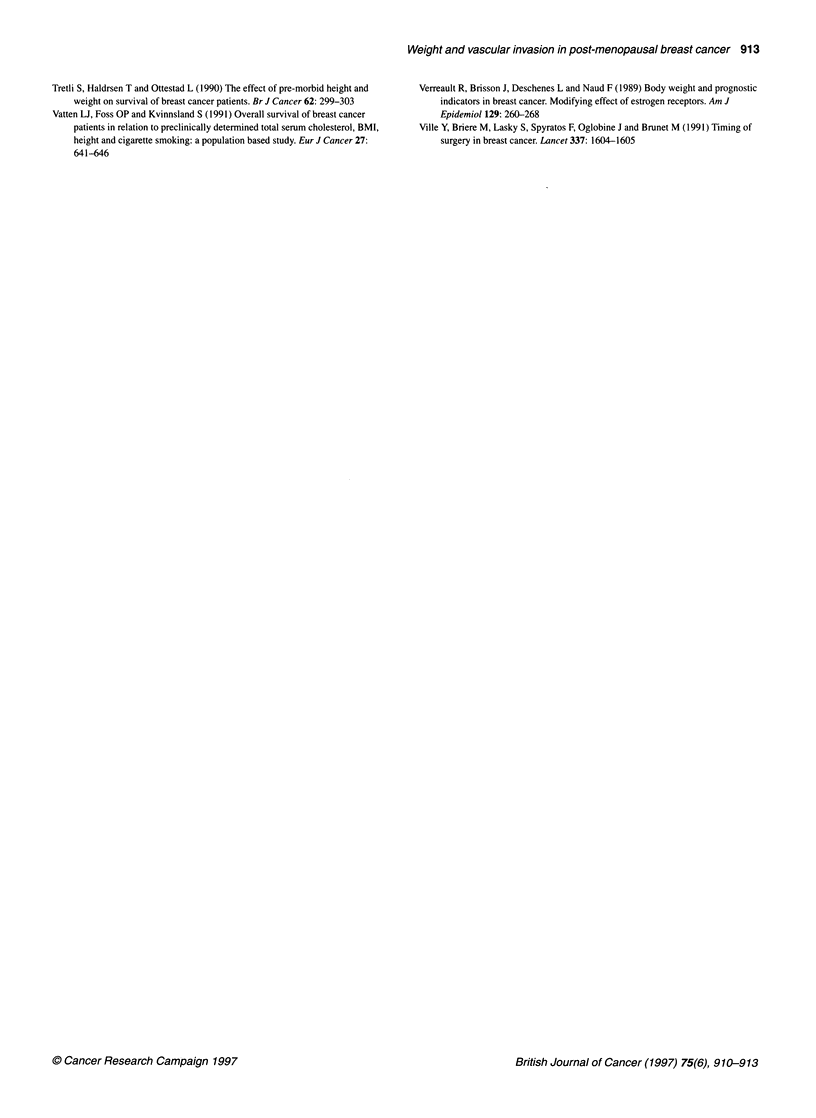# Body weight and vascular invasion in post-menopausal women with breast cancer.

**DOI:** 10.1038/bjc.1997.160

**Published:** 1997

**Authors:** R. A. Badwe, I. S. Fentiman, R. R. Millis, W. M. Gregory

**Affiliations:** ICRF Clinical Oncology Unit, Guy's Hospital, London, UK.

## Abstract

To examine the relationship between body weight and vascular invasion (VI) around tumours in post-menopausal women with operable breast cancer, a retrospective study was conducted of 393 patients treated in a breast unit between 1987 and 1991. Weight was measured at the time of diagnosis. Vascular invasion was recorded as being present or absent. Vascular invasion was seen in slightly more of the 50 perimenopausal patients than in the 343 post-menopausal women (44% vs 36%). In the tumour specimens from post-menopausal patients weighing <50 kg, VI was observed in 11% compared with 45% of those weighing more than 80 kg (P= 0.02). Furthermore, the 5-year survival of those with VI was 74% compared with 91% for those without (P < 0.0001). Menopausal status and body weight may influence survival in patients with breast cancer, possibly as a result of the presence of unopposed circulating oestrogens at the time of surgery. Oestrogens may alter cohesiveness of breast cancer cells and modulate secretion of proteases, thereby influencing invasive potential. Excision of tumours in such an environment may have a deleterious impact on survival.


British Journal of Cancer (1997) 75(6), 910–913
© 1997 Cancer Research Campaign

### Body weight and vascular invasion in post-menopausal women with breast cancer

**RA Badwe, IS Fentiman, RR Millis and WM Gregory**

ICRF Clinical Oncology Unit, Guy's Hospital, London SE1 9RT, UK

**Summary** To examine the relationship between body weight and vascular invasion (VI) around tumours in post-menopausal women with operable breast cancer, a retrospective study was conducted of 393 patients treated in a breast unit between 1987 and 1991. Weight was measured at the time of diagnosis. Vascular invasion was recorded as being present or absent. Vascular invasion was seen in slightly more of the 50 perimenopausal patients than in the 343 post-menopausal women (44% vs 36%). In the tumour specimens from post-menopausal patients weighing <50 kg, VI was observed in 11% compared with 45% of those weighing more than 80 kg ($P = 0.02$). Furthermore, the 5-year survival of those with VI was 74% compared with 91% for those without ($P < 0.0001$). Menopausal status and body weight may influence survival in patients with breast cancer, possibly as a result of the presence of unopposed circulating oestrogens at the time of surgery. Oestrogens may alter cohesiveness of breast cancer cells and modulate secretion of proteases, thereby influencing invasive potential. Excision of tumours in such an environment may have a deleterious impact on survival.

**Keywords:** breast cancer; prognosis; weight; post-menopausal women; vascular invasion

The prognosis of women with operable breast cancer depends upon tumour type, axillary nodal involvement and also menopausal status (Adami et al, 1986; Caleffi et al, 1989). Premenopausal cases have the lowest hazard rates for relapse and death. Rates are intermediate in post-menopausal women and the highest rates are found in perimenopausal women, that is those within 5 years of last menstrual period (Langlands et al, 1989). The observed differences may be due to the different hormonal environment to which tumours are exposed in these three groups of women.

Survival has been shown to be inversely related to body weight in the majority of studies (Boyd et al, 1981; Tartter et al, 1981; Newman et al, 1986; Tretli et al, 1990; Senie et al, 1991*a*; Vatten et al, 1991), but such a relationship is less obvious in premenopausal women (Greenberg et al, 1985). Although there is a relation between body weight and other prognostic factors, such as tumour size and axillary lymph node involvement, the effect of obesity remains an independent factor in multivariate analysis (Tarttar et al, 1981; Vatten et al, 1991). The mechanism through which menopausal status and body weight influence survival is uncertain, although it is known that most circulating oestrogens in post-menopausal women are derived from peripheral aromatization of adrenal androgens in subcutaneous fat. Thus, more obese women have higher plasma levels of oestrogens (Grodin et al, 1973). Additionally, the impact of obesity on survival is more pronounced among those women with oestrogen receptor-positive breast cancers (Verreault et al, 1989).

The risk of relapse and death also depends upon the metastatic potential of the tumour as manifested by the presence of malignant cells in lymphatic channels and blood vessels (vascular invasion, VI), both in and round the primary lesion (Bettelheim et al, 1984; Davis et al, 1985). For this reason, the relationship between weight, height and vascular invasion has been examined in a series of post-menopausal women with operable breast cancer.

*Received 10 June 1996*
*Revised 1 October 1996*
*Accepted 4 October 1996*

*Correspondence to:* IS Fentiman

### MATERIALS AND METHODS

A consecutive series of post-menopausal women with operable breast cancer treated at Guy's Hospital between 1987 and 1991 was studied. They were divided into two groups, perimenopausal and post-menopausal. Perimenopausal women were defined as those within 5 years of the last menstrual period (LMP). All patients beyond 5 years of the LMP were grouped as post-menopausal. Between 1987 and 1991, 460 such cases were seen, but 60 were excluded from the analysis because they were on steroid hormone treatment at the time of diagnosis. Data concerning body weight and height were not available in seven cases. Out of 393 eligible patients, 50 were perimenopausal and 343 were post-menopausal.

Height and weight were measured in metres and kilograms at the time of diagnosis in all patients. Those with a primary tumour < 4 cm were usually treated by a breast conservation procedure, whereas those with larger tumours underwent a modified radical mastectomy. Information on other prognostic factors such as tumour size, type, grade and nodal involvement was available from a computerized database.

Vascular invasion (VI) was defined as the presence of malignant cells within an endothelium-lined space in or around a primary breast cancer. No attempt was made to distinguish between lymphatics and small blood vessels. The majority of slides were reviewed by one pathologist (RRM) or by others under her direct supervision. Haematoxylin and eosin-stained sections of formalin-fixed tissue were examined and, in a few cases, methacarn-fixed

**Figure 1** Overall survival of patients with or without vascular invasion. $\chi^2_1$ = 17.54, $P < 0.001$

**Table 1** Prognostic variables in perimenopausal and post-menopausal women with operable breast cancer

| | Perimenopausal (*n* = 50) | Post-menopausal (*n* = 343) |
|---|---|---|
| Mean age + s.d. (years) | 52.6 + 3.7 | 64.0 + 7.6** |
| Mean tumour size + s.d. (cm) | 2.5 + 1.7 | 3.0 + 1.5* |
| Axillary nodal involvement (%) | 26 (52) | 152 (44) |
| Mean number of nodes + s.d. | 3.2 + 6.2 | 2.5 + 6.1 |
| Tumour type (%) | | |
| Ductal | 37 (74) | 244 (71) |
| Lobular | 3 (6) | 47 (14) |
| Other | 10 (20) | 52 (15) |
| Ductal tumour grade (% of ductal) | | |
| I | 5 (14) | 37 (15) |
| II | 14 (38) | 122 (50) |
| III | 18 (49) | 85 (35) |

Differences not significant unless shown. *$P$ = 0.04, Mann–Whitney test. **$P$ = << 0.001, Mann–Whitney test.

**Table 2** Logistic regression analysis for predictability of VI

| Variable (coding) | *Z*-value[a] | *P*-value | OR[b] |
|---|---|---|---|
| Nodes (0 vs 1–3 vs > 3) | 5.4 | <<0.0001 | 5.2 |
| Weight (continuous) | 4.1 | <0.0001 | 5.0 |
| Histology[c] (grade 1 vs 2 vs 3) | 2.7 | 0.008 | 2.9 |
| Height (continuous) | 2.2 | 0.024 | 2.4 |

[a]Standardized normal deviate. [b]Odds ratio – comparing extremes, i.e. 0 nodes vs >3 nodes, 50 kg vs 90 kg, grade 1 vs grade 3, 1.5 m vs 1.7 m. [c]Non-ductal histologies included with grade 2. The following factors were included in the analysis but did not reach statistical significance (P = 0.05): menstrual status, tumour size and patient age.

**Table 3** Univariate analysis of prognostic variables and survival

| Variable | Chi-square | *P*-value |
|---|---|---|
| Menopausal status | 3.6 | 0.58 |
| Age | 0.24 | 0.63 |
| Tumour size | 7.58 | 0.0059 |
| Histological type/grade | 8.12 | 0.0044 |
| Axillary nodal status | 12.07 | 0.0005 |
| Vascular invasion | 16.39 | 0.0001 |
| Body mass index | 0.58 | 0.45 |

tissue was also available. Between one and five slides were examined, depending on the size of the tumour. Suspicious areas were always examined under high power for confirmation of the presence of vascular invasion. Only unequivocal foci were accepted.

### Statistical methods

Non-parametric tests were used where possible. Correlation coefficients, where given, are Spearman's rank correlation coefficients. Logistic regression analysis was used to investigate multivariate correlates of vascular invasion. Survival curves were drawn using the method of Kaplan and Meier (1985), with significance being determined using the log-rank test (Peto et al, 1977). Multivariate survival analysis used Cox's proportional hazards model (Cox, 1976).

### RESULTS

The distribution of conventional prognostic factors in perimenopausal and post-menopausal cases was not significantly different, as shown in Table 1, with the exception that mean tumour size was slightly larger in post-menopausal patients. Vascular invasion was found in a greater proportion of perimenopausal patients than in post-menopausal cases (44% vs 36%), although this did not reach statistical significance ($P$ = 0.34). There was no significant relation between weight and vascular invasion (VI) in perimenopausal women. In post-menopausal patients, median weight for the group with vascular invasion was 69 kg (range 46–120 kg), which was significantly greater than that of the group without VI (63 kg, range 41–102 kg), $P < 0.0001$ (Mann–Whitney test). Among lighter women with weight < 50 kg, VI was present in 11% whereas, for heavier women weighing > 80 kg, it was observed in 45% of cases (Fisher's exact test, $P$ = 0.02).

Height was not correlated univariately with vascular invasion and showed only a weak multivariate correlation, after allowance for weight (Table 2). Further analysis using body mass index did not show a greater correlation than the analysis using weight alone. Multivariate logistic regression analyses demonstrated a further strong correlation of VI with the number of lymph node metastases and a weaker correlation with histological grade (Table 2). There was no significant relationship between oestrogen receptor (ER) of the primary tumour and vascular invasion in the whole group (Spearman rank correlation $r$ = 0.098, $P$ = 0.075) and, similarly, in the post-menopausal cases only a weak relationship ($r$ = 0.12, $P$ = 0.035). If ER was included in the Cox model, it was an independent prognostic factor but, because of the number of patients with unknown ER ($n$ = 60), there was a significant reduction of patients included within the model from 380 without ER to 325 with ER included.

Univariate and multivariate predictors of survival are shown in Table 3. Weight had no impact on survival. Vascular invasion was the strongest predictor. Five-year survival for women with VI was 74% compared with 91% for those without VI ($\chi^2$ = 17.5, $P < 0.0001$). When the Cox model was repeated, omitting VI, weight was not a prognostic indicator in univariate or multivariate analysis.

### DISCUSSION

This study has shown that there is a highly significant relationship between weight and vascular invasion in and around breast cancers in post-menopausal women. Heavier women are exposed to higher levels of oestrogens as a result of peripheral aromatization of androgen (Grodin et al, 1973; Hemsell et al, 1974). It is possible that the observed increase in rate of vascular invasion may be due to an effect of oestrogen on the primary tumour. It has been shown previously that premenopausal women exposed to unopposed oestrogen at the time of tumour excision have a worse prognosis than that of women undergoing surgery during the luteal phase of the menstrual cycle (Badwe et al, 1991). The magnitude of the effect in premenopausal women was similar in oestrogen receptor-positive ($ER^+$) and -negative ($ER^-$) cases, suggesting that this might result from activation of $ER^+$ peritumoral normal tissue. Similarly, in this study the ER status of the tumours did not have any impact on vascular invasion or prognosis.

There is still no uniformity concerning these findings, which have been confirmed by some (Hrushesky et al, 1989; Senie et al, 1991*b*; Ville et al, 1991) but not by others (Goldhirsch et al, 1991; Low et al, 1991; Powles et al, 1991). However meta-analysis of published studies has shown an overall significant effect of timing of surgery on both relapse-free and overall survival (Fentiman et al, 1994). Furthermore, a recent study showed that vascular invasion was observed in 47% of tumours excised between days 3 and 12 of the cycle but in only 33% of those undergoing surgery at other times (Badwe et al, 1995). In vitro, oestrogens modulate secretion of at least two proteases, plasminogen activator (Mira-y-Lopez et al, 1991) and cathepsin D (Rochefort et al, 1990) which can activate a cascade of proteolysis (He et al, 1989). The ability to secrete these proteases has been shown to be an independent adverse predictor of survival in breast cancer (Rochefort, 1990; Duffy et al, 1991).

Perimenopausal women experience infrequent ovulation and those with breast cancers are more likely to be exposed to unopposed oestrogens at the time of surgery. Circulating oestradiol levels in perimenopausal women are variable but can reach premenopausal follicular levels. Once the ovaries have ceased to produce active hormones, the major source of oestrogen is body fat. It has been reported that women with breast cancer aged 50 years or over who have four or more axillary lymph node metastases were more likely to be obese (Daniell et al, 1988).

Thus, unopposed oestrogens may induce secretion of proteases which allow a more dyscohesive tumour to gain access to vascular channels and hence attempts at surgical excision of the tumour may be deleterious in such an environment. The data from this study imply that tumour behaviour in post-menopausal women may be modulated by oestrogens. Additionally, they suggest avenues for further research with preoperative hormonal manipulations in studies with both biological and clinical end points.

### ACKNOWLEDGEMENT

This work was funded by the Imperial Cancer Research Fund.

### REFERENCES

Adami OH, Malker B, Holmberg L, Persson I and Stone B (1986) The relation between survival and age at diagnosis in breast cancer. *N Engl J Med* **315**: 559–563

Badwe RA, Gregory WM, Chaudary MA, Richards MA, Bentley AE, Rubens RD and Fentiman IS (1991) Timing of surgery during the menstrual cycle and survival of premenopausal women with operable breast cancer. *Lancet* **337**: 1261–1264

Badwe RA, Bettelheim R, Millis RR, Gregory WM, Richards MA and Fentiman IS (1995) Cyclical variations in premenopausal women with early breast cancer. *Eur J Cancer* **31A**: 2181–2194

Bettelheim R, Penman HG, Thornton-Jones H and Neville AM (1984) Prognostic significance of peritumoral vascular invasion in breast cancer. *Br J Cancer* **50**: 771–777

Boyd NF, Campbell JE, Germanson T, Thomson DB, Sutherland DB and Meakin JW (1981) Body weight and prognosis in breast cancer. *J Natl Cancer Inst* **67**: 785–789

Caleffi M, Fentiman IS and Birkhead BG (1989) Factors at presentation influencing the prognosis in breast cancer. *Eur J Cancer Clin Oncol* **25**: 51–56

Cox DR (1972) Regression models and life tables. *J R Stat Soc (B)* **34**: 187–220

Daniell HW (1988) Increased lymph node metastases at mastectomy for breast cancer associated with host obesity, cigarette smoking, age and large tumour size. *Cancer* **62**: 429–435

Davis BW, Gelber R, Goldhirsch A, Hartmann WH, Hollaway L, Russell I and Rudenstam CM (1985) Prognostic significance of peritumoral vessel invasion in clinical trials of adjuvant therapy for breast cancer with axillary lymph node metastasis. *Hum Pathol* **16**: 1212–1218

Duffy MJ, Brouillet J-P, Reilly D, McDermott E, O'Higgins N, Fennelly JJ, Maudelonde T and Rochefort H (1991) Cathepsin-D concentration in breast cancer cytosols: correlation with biochemical, histological, and clinical findings. *Clin Chem* **37**: 101–104

Fentiman IS, Gregory WM, Richards MA (1994) Effect of menstrual cycle on surgical treatment of breast cancer. *Lancet* **344**: 402

Goldhirsch A, Gelber R, Forbes J, Price K, Castiglione M, Rudenstam C-M, Lindtner J, Hacking A and Senn H (1991) Timing of breast cancer surgery. *Lancet* **344**: 691–692

Greenberg ER, Vessey MP, McPherson K, Doll R and Yeats D (1985) Body size and survival in premenopausal breast cancer. *Br J Cancer* **51**: 691–697

Grodin JM, Siiteri PK and MacDonald PC (1973) Source of estrogen production in postmenopausal women. *J Clin Endocrinol Metab* **36**: 207–214

He CS, Wilhelm SM, Pentland AP, Marmer BL, Grant GA, Eisen AZ and Goldberg GI (1989) Tissue cooperation in a proteolytic cascade activating interstitial collagenase. *Proc Natl Acad Sci USA* **86**: 2632–2636

Hemsell DL, Grodin JM and Brenner PF (1974) Plasma precursors of estrogen. *J Clin Endocrinol Metab* **38**: 476–479.

Hrushesky WJ, Bluming AZ, Gruber SA and Sothern RB (1989) Menstrual influence of surgical cure of breast cancer. *Lancet* **2**: 949–952

Kaplan EL and Meier P (1958) Nonparametric estimation from incomplete observations. *Am Stat Assoc J* **53**: 457–481

Langlands AO, Pocock SJ, Kerr GR and Gore SM (1979) Long term survival of patients with breast cancer: a study of the curability of the disease. *Br Med J* **2**: 1247–1251

Low SC, Galea MA and Blamey RW (1991) Timing of breast cancer surgery *Lancet* **338**: 691–692

Mira-y-Lopez R, Osborne MP, DePalo AJ and Ossowski L (1991) Estradiol modulation of plasminogen activator production in organ culture of human breast cancer. *Int J Cancer* **47**: 827–832

Newman SC, Miller AB and Howe GR (1986) A study of the effect of weight and dietary fat on breast cancer survival time. *Am J Epidemiol* **123**: 767–773

Peto R, Pike MC, Armitage P, Breslow NE, Cox DR, Howard SV, Mantel N, McPherson K, Peto J and Smith PG (1977) Design and analysis of randomised clinical trials requiring prolonged observation of each patient. II. Analysis and examples. *Br J Cancer* **35**: 1–39

Powles TJ, Ashley SE, Nash AG, Tidy A, Gazet J-C and Ford HT (1991) Timing of surgery in breast cancer. *Lancet* **337**: 1604

Rochefort H (1990) Cathepsin-D in breast cancer. *Breast Cancer Res Treat* **16**: 3–13

Senie RT, Rosen PP, Rhodes P, Lesser ML and Kinne DW (1991*a*) Obesity at diagnosis of breast cancer influences duration of disease free survival. *Ann Int Med* **116**: 26–32

Senie RT, Rosen PP, Rhodes P and Lesser ML (1991*b*) Timing of breast cancer excision during menstrual cycle influences duration of disease-free survival. *Ann Int Med* **115**: 337–342

Tartter PI, Papatestas AE, Ioannovich J, Mulvihill MN, Lesnick G and Aufses AH (1981) Cholesterol and obesity as prognostic factors in breast cancer. *Cancer* **47**: 2222–2227

© Cancer Research Campaign 1997

Tretli S, Haldrsen T and Ottestad L (1990) The effect of pre-morbid height and weight on survival of breast cancer patients. *Br J Cancer* **62**: 299–303

Vatten LJ, Foss OP and Kvinnsland S (1991) Overall survival of breast cancer patients in relation to preclinically determined total serum cholesterol, BMI, height and cigarette smoking: a population based study. *Eur J Cancer* **27**: 641–646

Verreault R, Brisson J, Deschenes L and Naud F (1989) Body weight and prognostic indicators in breast cancer. Modifying effect of estrogen receptors. *Am J Epidemiol* **129**: 260–268

Ville Y, Briere M, Lasky S, Spyratos F, Oglobine J and Brunet M (1991) Timing of surgery in breast cancer. *Lancet* **337**: 1604–1605

© Cancer Research Campaign 1997